# High-performance PTFE composites from industrial scrap with enhanced strength and wear resistance

**DOI:** 10.1038/s41598-025-13643-7

**Published:** 2025-08-04

**Authors:** Ahmed Kamal, Ahmed O. Mosleh, Ahmed Gaafer, Rasha A. Youness, Mohammed A. Taha

**Affiliations:** 1 Mechanical Engineering Department, Shoubra Faculty of Engineering, Benha University, 108 Shoubra St, Banha, Egypt; 2 Spectroscopy Department, National Research Centre, El Buhouth St, Dokki, Giza, 12622 Egypt; 3 Solid State Physics Department, National Research Centre, El Buhouth St, Dokki, 12622 Giza Egypt

**Keywords:** PTFE waste, Recycling, CTE, Mechanical properties, Wear resistance, Powder metallurgy, Structural materials, Composites

## Abstract

Due to the high cost of raw materials, this work aims to utilize polytetrafluoroethylene (PTFE) scrap generated from industrial waste to produce composites possessing superior properties for potential use in various industrial applications. In this respect, PTFE-based composites reinforced with mono- and hybrid granite and boron carbide (B_4_C) nanoparticles are produced using powder metallurgy (PM) technology. The sintered composites’ physical, mechanical, tribological, and thermal properties and the phase composition and microstructure were investigated using X-ray diffraction (XRD) and field emission scanning electron microscopy (FESEM) techniques, respectively. The results indicated that the phase composition of the prepared composites did not change. Adding granite and/or B_4_C to the PTFE base increased the bulk density and the total porosity, while the relative density decreased. In addition, after adding 5 vol% granite/5 vol% B_4_C (PTFE6 sample), there was a clear improvement in mechanical properties, including microhardness, ultimate, and Young’s modulus, reaching 123.29%, 91.33%, and 74.17% compared with the unreinforced sample (PTFE0). Moreover, there was a noticeable improvement in the wear rate, fraction coefficient, and thermal expansion coefficient (CTE) value for the same sample, which decreased by approximately 37.17%, 36.50%, and 61.64%.

## Introduction

Polytetrafluoroethylene (PTFE) is recognized as a critical material across industries due to its unique properties, including high chemical resistance, non-toxicity, and thermal stability, which can withstand both high and low temperatures, ranging from − 200 to + 260 °C. These attributes make it indispensable in aerospace, automotive, electronics, and chemical processing^1,2^. However, PTFE’s environmental impact is significant, as its resistance to degradation contributes to long-term waste accumulation. This challenge has driven extensive research into sustainable solutions, including recycling PTFE and enhancing its performance through reinforcement with advanced materials^3,4^. Recently, studies have focused on recycling PTFE waste to support sustainability initiatives, reduce environmental pollution, and promote a circular economy. Recycling PTFE reduces industrial waste and maintains the material’s beneficial properties, making it reusable for the applications mentioned earlier^5,6^. PTFE in its pure form cannot be used in various applications due to its poor mechanical properties, high wear resistance, and high CTE.

These weak properties can be improved to make composites based on PTFE and reinforced with other materials such as metals, nanofillers, carbon, and ceramics^7–10^. Reinforcement’s mechanical, thermal, and tribological properties depend on fillers’ type, size, amount, preparation, and process factors. Among the most used ceramics reinforcements are aluminium oxide (Al_2_O_3_), silicon carbide (SiC), graphene, ultrafine glass fibres, and bronze particles^11–16^. B_4_C is very likely the most crucial non-oxide ceramic. It has a wide range of desirable qualities, including excellent oxidation, high strength, outstanding chemical erosion resistance, thermal stability, and a high melting point, making it a good choice as a reinforcement to improve the properties of PTFE^17^. Moreover, granite dust, a ceramic byproduct derived from granite rocks, is a potential material for reinforcing and enhancing the aforementioned qualities of PTFE. This assignment was selected because of its superior hardness, strength, modulus, cost-effectiveness, and lightweight properties. Granite dust mainly consists of silicon oxide (SiO_2_) and Al_2_O_3_^18^.

The PM method, which involves powder mixing, consolidation, and hot-press sintering, is one of the best methods for preparing PTFE-based composites^19–21^. Compared to more conventional approaches, PM has several benefits when preparing composites based on PTFE. In addition to enabling the production of complicated forms, PM makes it possible to manufacture near-net-shape or net-shape products, reducing material waste and the expenses associated with machining. In addition, PM allows for more control over porosity, the reinforcement in the matrix is more uniform, the process temperature is lower, and it may generate composites with mechanical, tribological, and thermal characteristics that can be tuned to the specific needs of the material. It is possible that traditional processes, such as machining from bulk material, are less efficient, resulting in a greater amount of waste and possibly restricting the intricacy of the final product. It is worth noting that the limitations of powder metallurgy technology are that it requires advanced machinery, such as a furnace mill with a constant heating rate. In addition, appropriate factors such as mixing factors, sintering temperature, and heating rate must be used for each material, and this depends on experience^22–27^.

Many researchers have attempted to add suitable reinforcements to the PTFE matrix to improve the desired properties, such as mechanical properties, corrosion resistance, etc. For example, Chao et al.^28^ used a cold pressing and sintering technique to incorporate Al₂O_3_ particles into a PTFE matrix. It examined how the mass content of the Al₂O_3_ platelets affected the composites’ mechanical characteristics, thermal conductivity, and dielectric qualities. The results showed that the Al₂O_3_/PTFE composites improved strength and thermal conductivity compared to PTFE. Liu et al.^29^ reported that polyimide and boron nitride combinations have enhanced tribological performance. Sharma et al.^30^ studied bronze and nano-Al₂O₃ reinforcements that have improved wear resistance under varying operational conditions. Shi et al.^31^ indicated that the strength of PTFE might be enhanced by including 1% carbon nanofibers.

In light of the above, there have been previous studies on the use of PTFE-based composites, using the traditional preparation method, but the novelty in this manuscript is the use of PM technology to maximize the utilization of PTFE scrap from industrial waste and reuse it as a basis for manufacturing dense composites with excellent mechanical and tribological properties, as well as thermal expansion. In addition, single and hybrid nanoparticles of granite waste and B_4_C particles were used as enhancers to improve the above-mentioned properties. Then, PTFE-based composites were prepared with different proportions of mono and hybrid granite up to 5 vol% and B_4_C up to 5 vol%, then pressed and sintered under 120 bar pressure. Moreover, the effect of adding hybrid reinforcement on microstructure, density, mechanical properties, CTE value, and wear resistance of prepared composites was investigated.

## Materials and experimental work

In this work, the methodology consisted of materials preparation, manufacturing, and characterization of the manufactured composite to produce the aimed composites.

### Materials

**Fig. 1:**
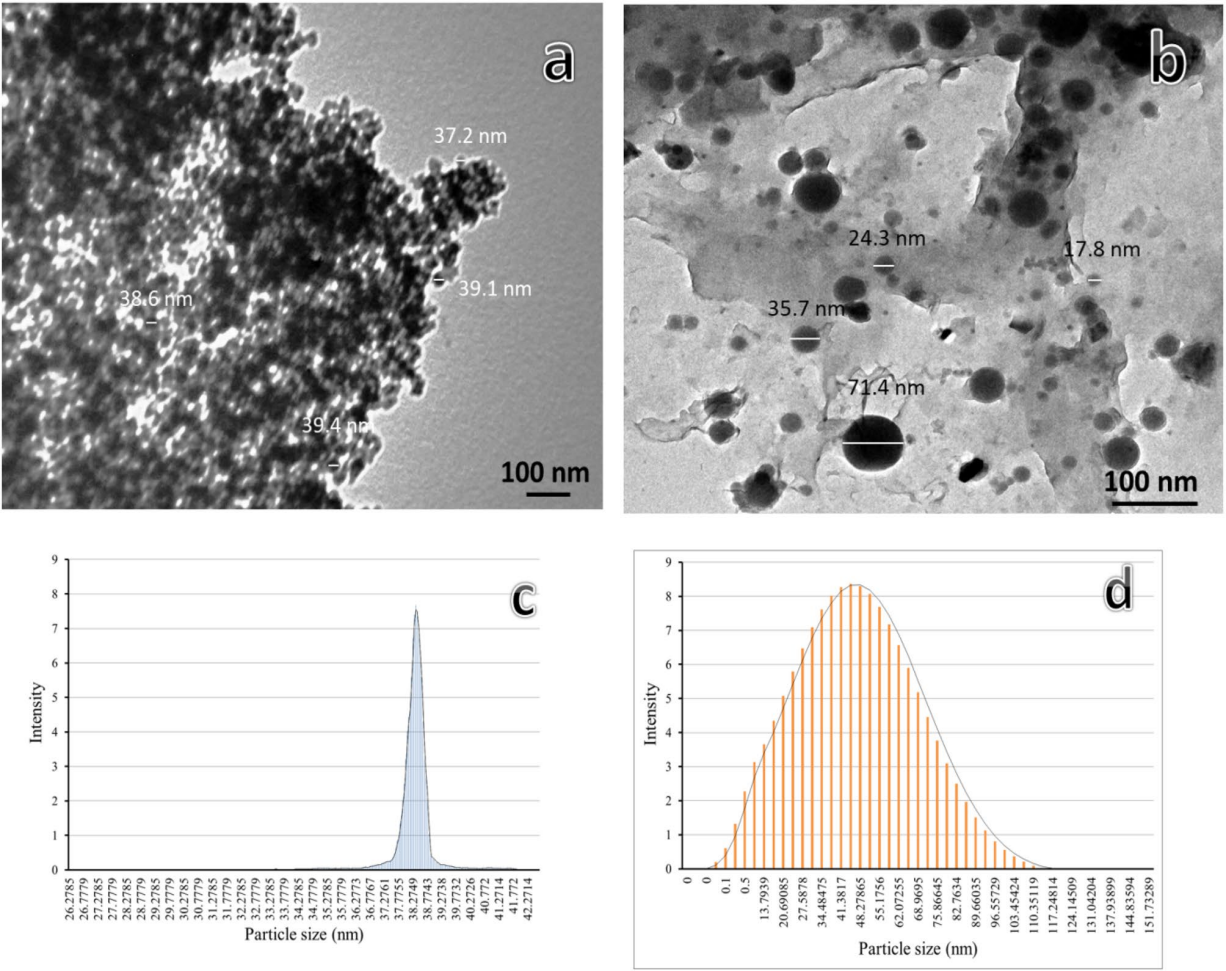
TEM micrographs and particle size distribution of B_4_C (**a**, **b**) and granite (**c**, **d**) nanoparticles.

The matrix material used in this study was PTFE scrap with a 2.200 g/cm^3^ density, while the reinforcements were granite waste and B_4_C powders with 2.680 and 2.520 g/cm^3^ densities, respectively. PTFE scrap was obtained from industrial waste, cleaned, dried, and ground into fine particles. According to transmission electron microscopy (TEM, type JEOL JEM-1230) and particle size distribution (a diffraction particle size analyzer), as shown in Fig. 1(a-d), the B_4_C powders appear as particles with more agglomerates and an average size of 38.58 nm. In contrast, granite appears as particles with low agglomerates and a size range of 17–71 nm. The composition of the granite waste powder is provided in Table 1.

**Table 1:** Composition of granite waste powder (wt.%).

Element	SiO_2_	Al_2_O_3_	Fe_2_O_3_	CaO	MgO	K_2_O	TiO_2_	Residue
wt.**%**	65.63	18.27	6.15	4.76	2.64	1.03	1.38	0.15

### Sample Preparation

#### Mixing

The homogenization of the raw materials, including PTFE, granite, and B_4_C, was performed using a ball mill for 10 h with a ball-to-powder ratio. (BPR) of 1:5 and 120 rpm as a rotation speed. Table 2 summarizes the batch design of the prepared composites, indicating the volumetric compositions for each sample.

**Table 2:** Batch design of the investigated prepared nanocomposites.

Samples name	Composition (Vol.%)		
	PTFE	B_4_C	granite
PTFE0	100	0	0
PTFE1	95	0	5
PTFE2	95	5	0
PTFE3	95	2.5	2.5
PTFE4	92.5	2.5	5
PTFE5	92.5	5	2.5
PTFE6	90	5	5

#### Mold design

**Fig. 2:**
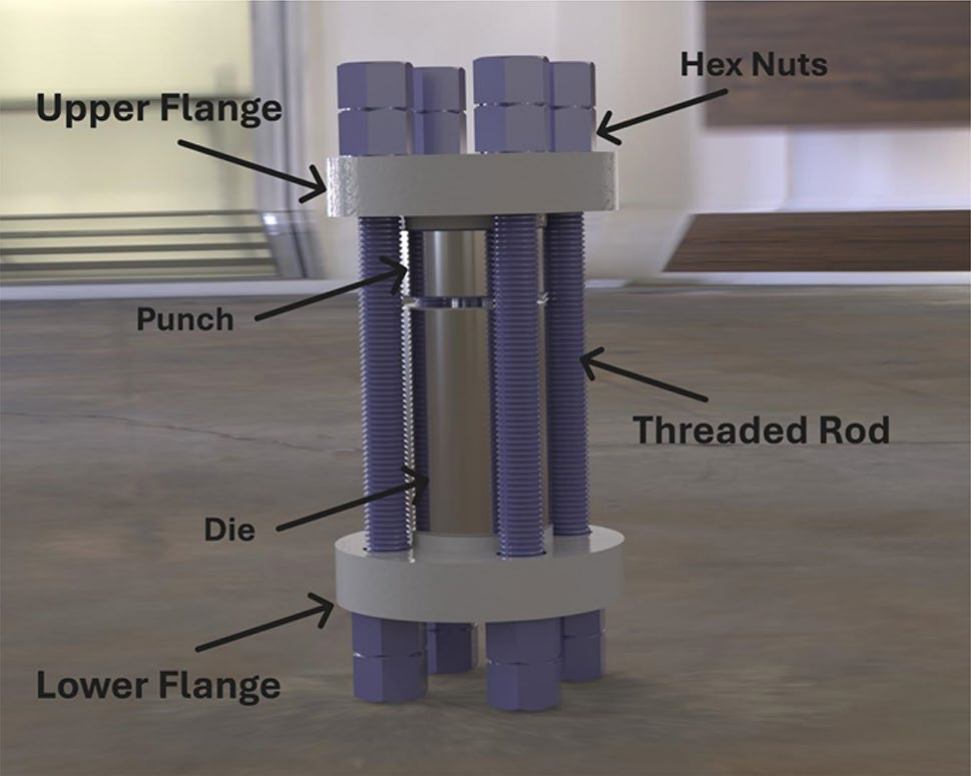
The image shows a mold assembly.

The mold assembly is shown in Fig. 2, which consists of several critical components designed to withstand the high pressures and temperatures involved in the composite manufacturing process. The upper and lower flanges serve as the main structural supports, maintaining alignment between the punch and die and ensuring even distribution of compressive forces during the pressing and sintering stages. The punch and die, located centrally, are the primary functional elements responsible for shaping and compacting the composite material. These components are made from high-strength steel to prevent deformation under operational loads. Threaded rods secure the assembly, providing axial support and firmly holding the flanges, punch, and die together. These rods are made from alloy steel, chosen for their ability to resist tensile stresses during manufacturing. The Hex Nuts at the top and bottom of the threaded rods are tightened to apply uniform pressure across the assembly. They are fabricated from materials with high yield strength to ensure stability and prevent loosening under repeated thermal cycles. This robust design ensures structural integrity, uniform pressure distribution, and proper venting of trapped gases during sintering, thereby minimizing defects such as voids or cracks in the final composite material.

#### Compaction and sintering

**Fig. 3:**
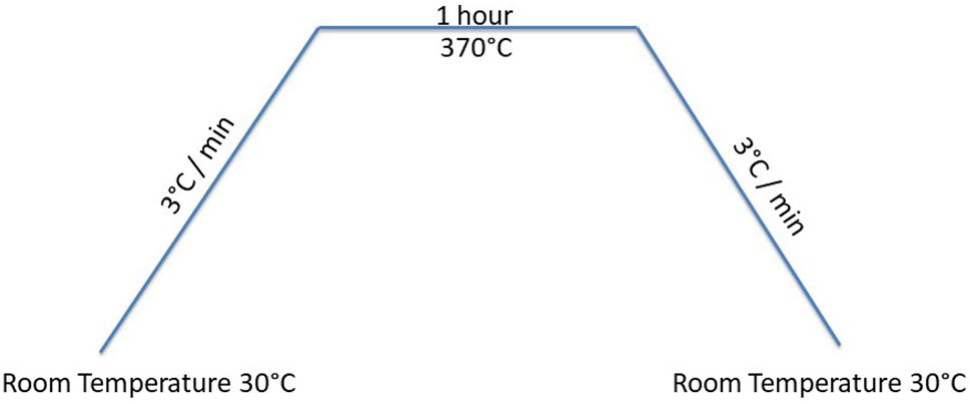
A heating cycle diagram of the programmable furnace.

**Fig. 4:**
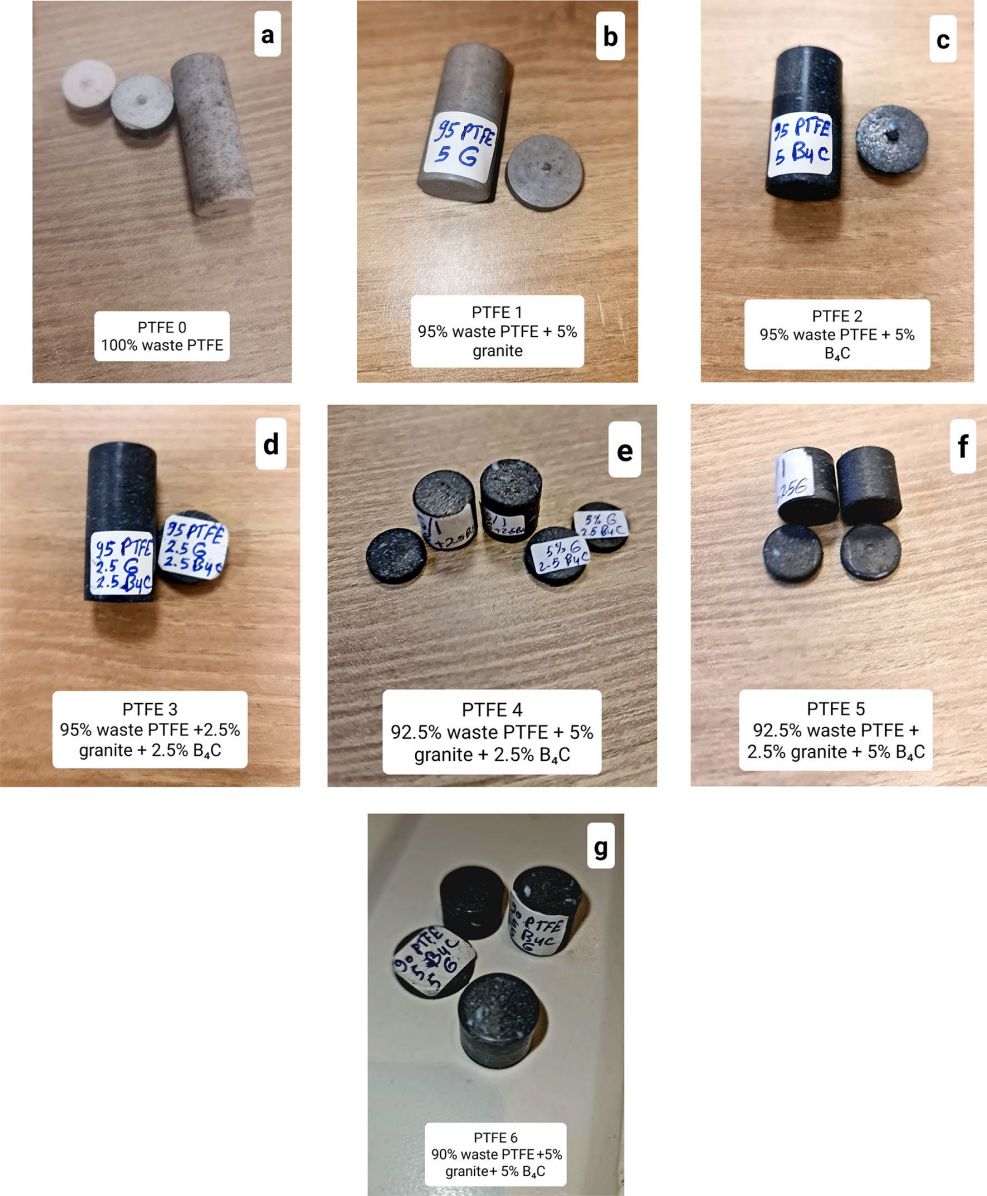
Images of samples after fabrication.

The compaction of the PTFE-based composite powders was conducted using a hydraulic press, as shown in Fig. 2. The powder was loaded into a steel cylindrical die with an internal diameter of 20 mm and a height of 80 mm and compressed using a steel punch that applied a uniaxial load vertically at room temperature. Each sample was pressed under a constant pressure of 120 bar (12 MPa) to ensure uniform densification. Compression was applied by manually tightening the hex nuts on the threaded rods, which gradually transferred force through the punch onto the powder. Once the powder was sufficiently compressed, the nuts were left fixed in place to maintain the applied pressure during the subsequent sintering stage, ensuring structural stability (Fig. 2). After that, a programmable furnace sintered the compacted samples (under pressure) at 370 °C for 1 h, with heating and cooling rates of 3 °C/min. During sintering, the clearance between the punch and die was adjusted to release trapped gases, preventing defects such as voids or cracks. Figures 3 and 4 show a heating cycle diagram of the programmable furnace and images of the prepared samples, respectively.

### Characterization of sintered samples

#### Morphology and phase composition

The phases of the sintered samples were detected using XRD (Philips PW) analysis. FESEM coupled with energy dispersive X-ray analysis **(**EDX) (type Quanta FEG250 with an accelerating voltage of 30 kV and a magnification of 10 × up to × 300,000) was used to examine the microstructure of sintered samples.

#### Physical properties

The Archimedes technique (ASTM: B962-13) examined the bulk density, total porosity, and relative density of PTFE and all composite samples.

#### Thermal properties

Netzsch DIL402 PC studied thermal expansion of samples in the range 25–200 °C with a heating rate of 5 °C/min using rectangular bars.

#### Mechanical investigation

The microhardness (Hv) of sintered samples was measured using a Vickers tester according to ASTM: B933-09 with a load (p) of 1.9 N for 5 s using Eq. 1^32^.

1 $$\:\text{H}\text{v}=1.854\:\text{x}\:\frac{\text{P}}{{\text{d}}^{2}}$$

Where d is the diagonal of indentation, the compressive tests of the sintered specimens were carried out at a strain rate of 1 mm/min.

Ultrasonic longitudinal (V_L_) and shear wave velocities (V_S_) were measured in the PTFE and its composites, using the pulse-echo technique system. The values of Lame’s constants are obtained from V _L_ and V_S_ as follows^33,34^

2 $$\:{\uplambda\:}={\uprho\:}({V}_{L}^{2}-2{\text{V}}_{S}^{2})$$

3 $$\:{\upmu\:}\:={\uprho\:}{V}_{S}^{2}\:$$

The longitudinal modulus (L), Young’s modulus (Y), shear modulus (G), bulk modulus (B), and Poisson’s ratio (ν) of the PTFE and its composites were calculated according to the formula^35^:

4 $$\:L=\lambda\:+2\mu\:\:$$

5 $$\:G=\mu\:$$

6 $$\:E=\mu\:\frac{3\lambda\:+2\mu\:}{\lambda\:+\mu\:}$$

7 $$\:B=\lambda\:+\frac{2}{3}\mu\:$$

8 $$\upsilon = \frac{\lambda }{{2\left( {\lambda + \mu } \right)}}$$

#### Tribology investigation

The pin-on-disc wear test was performed using a TNO tester (Delft, The Netherlands) based on the ASTM G99-04 A standard at room temperature. The process parameters of the wear test involved a speed of 0.25 m/s, a sliding time of 300 s, and different applied loads of 5, 10, and 20 N. The following Eqs. (9) and (10) were used to calculate the wear rate of sintered samples^36^.

9 $${\text{Weight loss }} = {\text{ weight before wear }} - {\text{ weight after wear}}$$

10 $$\:\text{W}\text{e}\text{a}\text{r}\:\text{r}\text{a}\text{t}\text{e}=\:\frac{\text{W}\text{e}\text{i}\text{g}\text{h}\text{t}\:\text{l}\text{o}\text{s}\text{s}}{\text{S}\text{l}\text{i}\text{d}\text{i}\text{n}\text{g}\:\text{t}\text{i}\text{m}\text{e}}$$

**Fig. 5:**
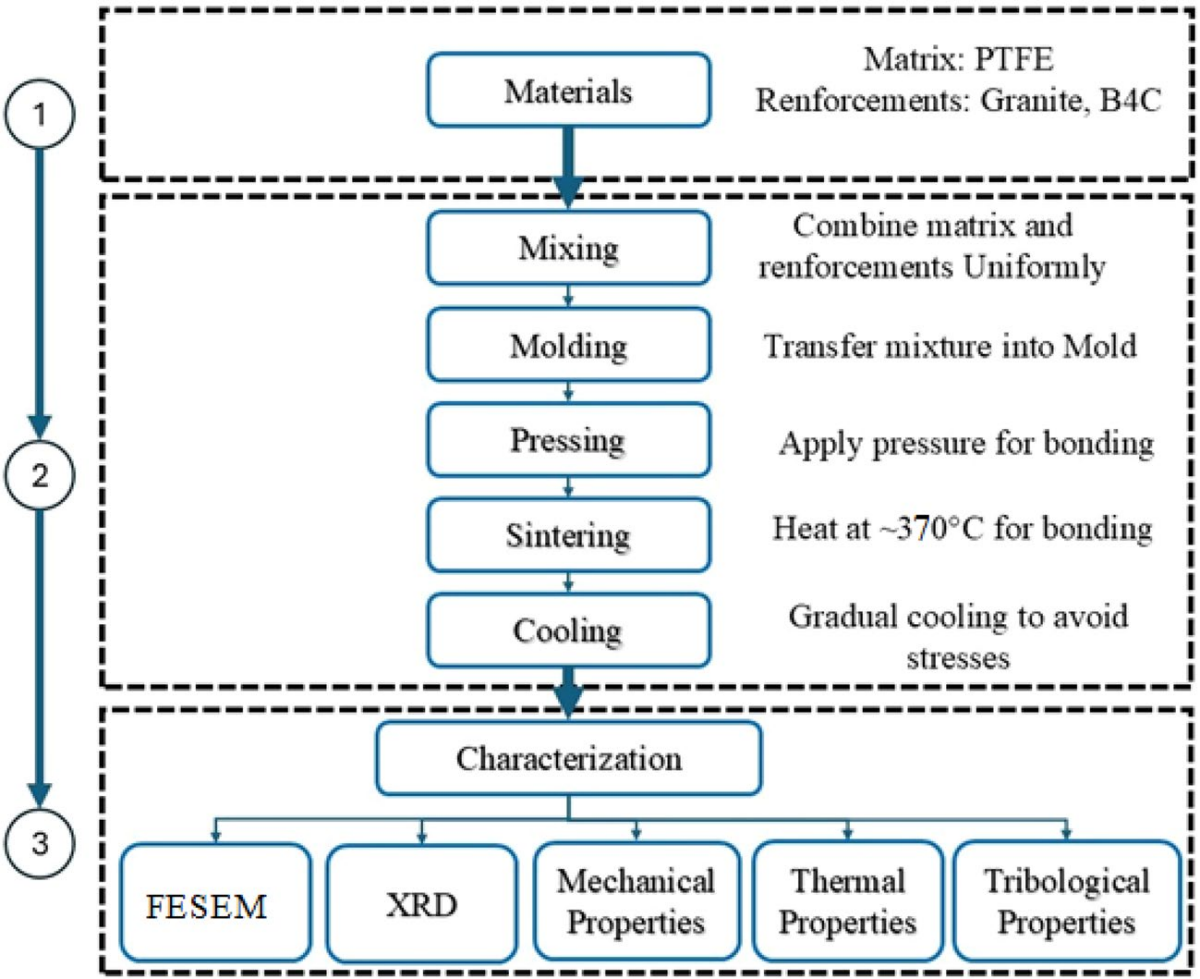
Schematic of methodology for PTFE composite fabrication and characterization.

The flowchart of the work methodology used in the current work is shown in Fig. 5.

## Results and discussion

### Phase composition

**Fig. 6:**
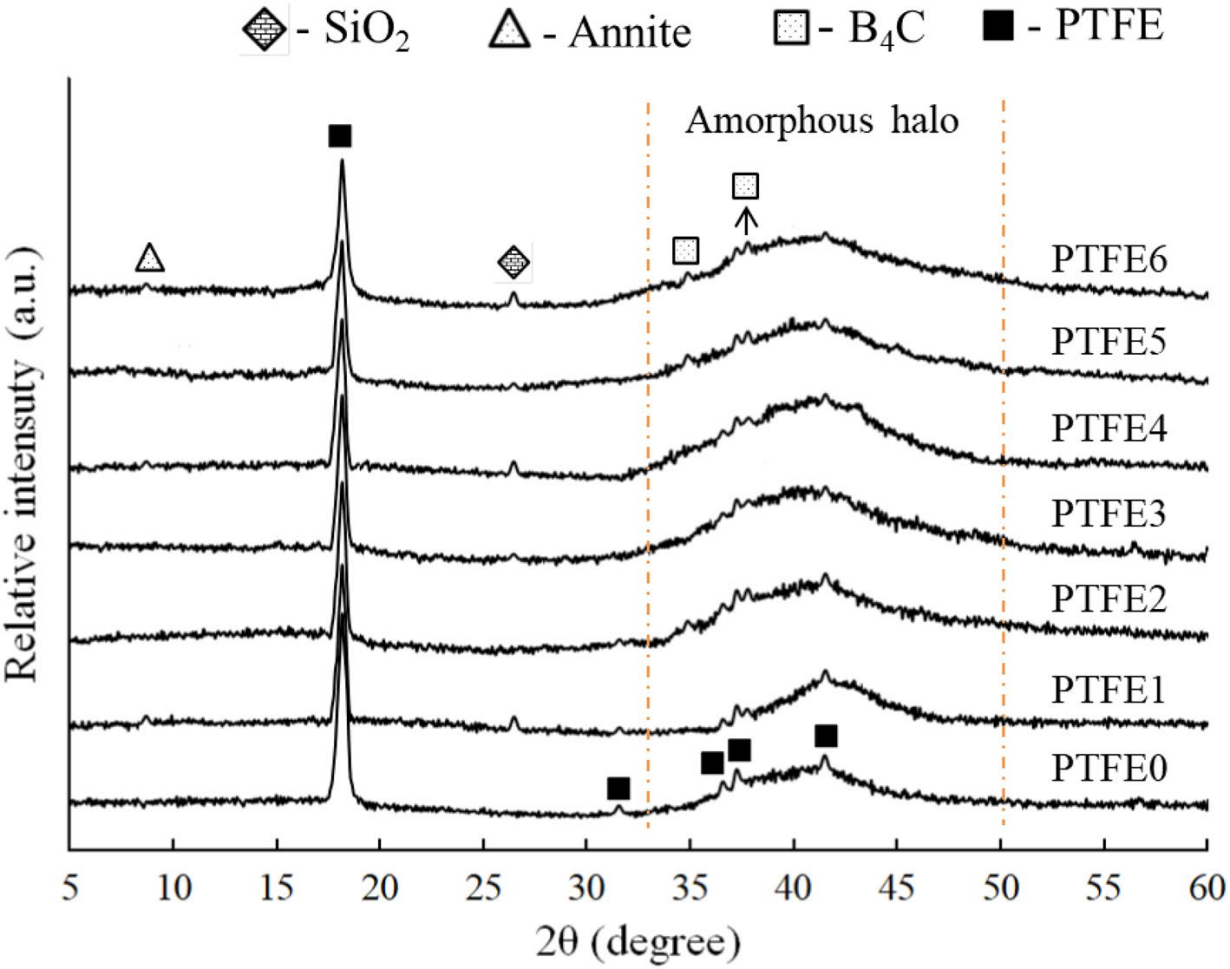
XRD patterns of sintered PTFE matrix composite containing mono and hybrid granite and B_4_C reinforcements.

The XRD patterns of sintered PTFE and its composites are shown in Fig. 6. For the base (PTFE0) sample, the XRD patterns were analyzed according to JCPDS 47-2217 standard cards and Refs^37,38^. appear mainly composed of PTFE. It can be seen that there is a strong diffraction peak appearing at 2θ = 18.10⁰, and four other weak peaks appearing at 2θ = 31.60⁰, 36.64⁰, 37.13⁰, and 41.41⁰ superimposed on an amorphous halo (broad peak under the crystalline peaks) around 40⁰. Moreover, for composite samples, according to JCPDS 35–0798, B_4_C appears in phase at 2θ = 37.82⁰ and 34.96⁰ while SiO_2_ and annite phases appear at 2θ = 26.62⁰ and 8.78⁰ according to JCPDS 88-2487 and 42-1413, respectively.

### Microstructural analysis

**Fig. 7:**
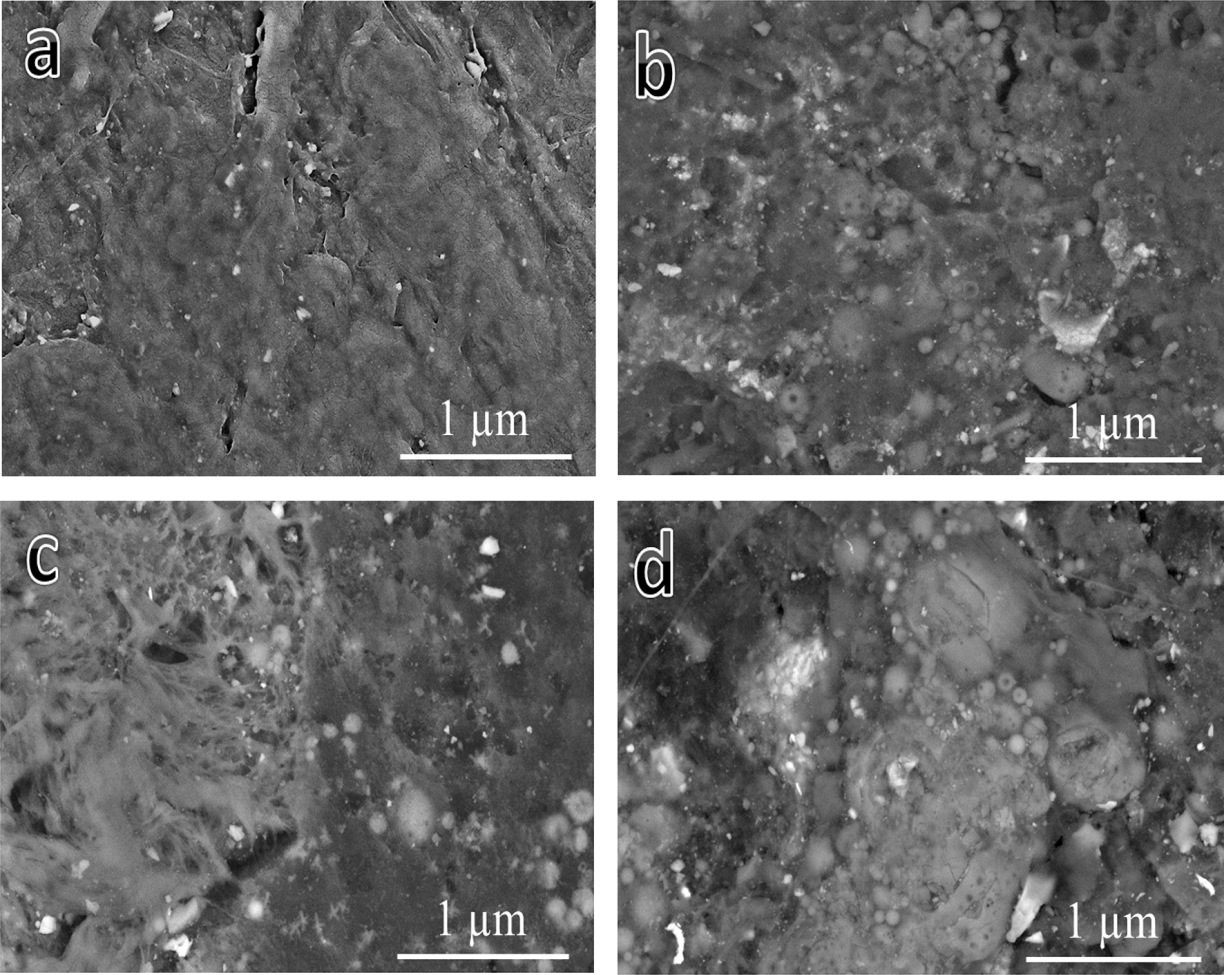
FESEM images of sintered (**a**) PTFE0, (**b**) PTFE1, (**c**) PTFE2, and (**d**) PTFE6 samples.

**Fig. 8:**
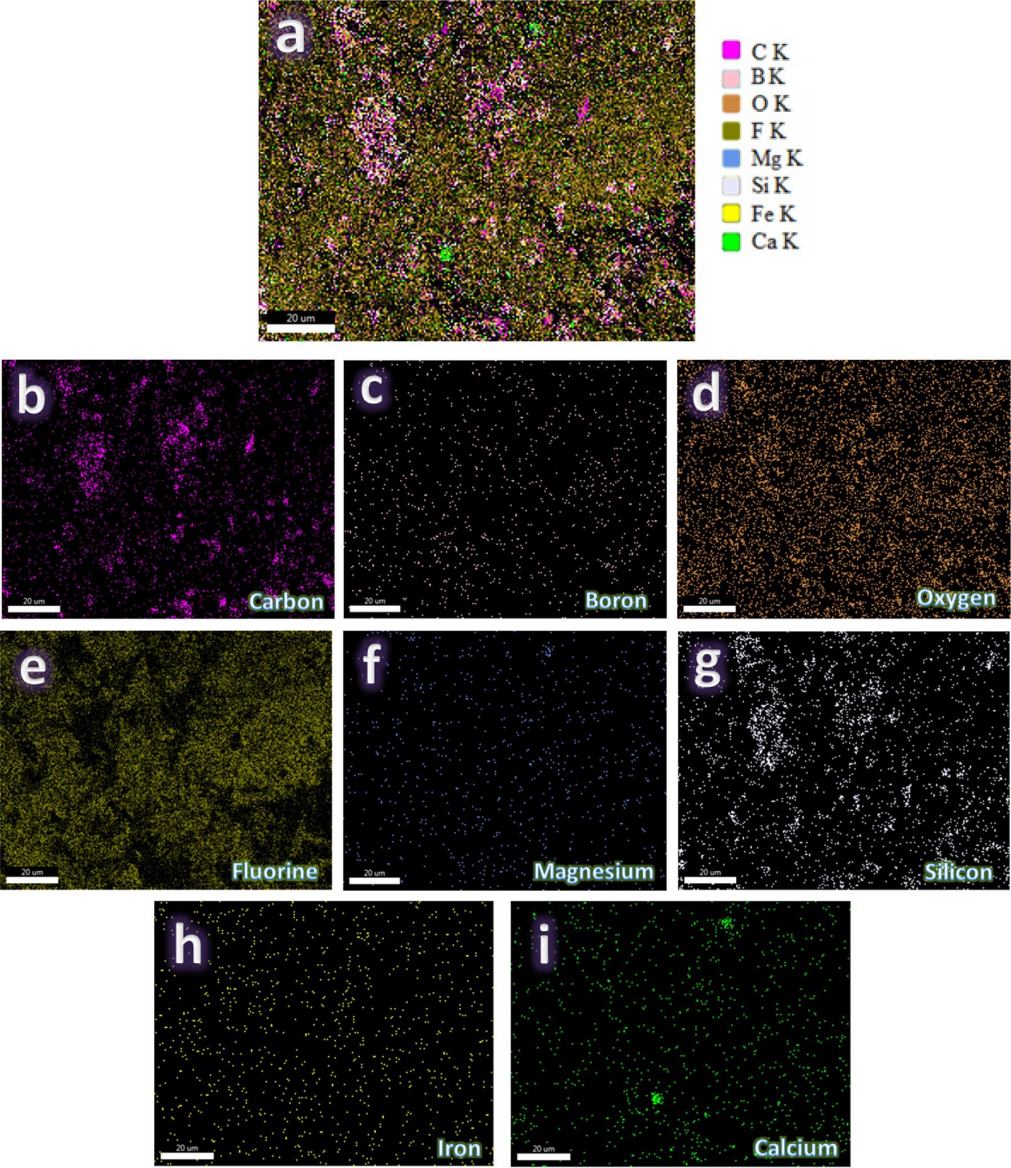
(**a**) EDX mapping of all constituents in the PTFE6 sample and the EDX mapping of the distribution of each component, i.e., (**b**) carbon, (**c**) boron, (**d**) oxygen, (**e**) fluorine, (**f**) magnesium, (**g**) silicon, (**h**) iron, and (**i**) calcium.

Figure 7(a-d) shows the microstructural characterization of sintered waste PTFE composites, which was conducted using FESEM to evaluate the effect of reinforcement on the matrix. Figure 6a represents 100% waste PTFE and shows a smooth and homogenous surface with minimal defects, serving as a baseline for comparison. Figure 6b, showcasing the 95% waste PTFE and 5% granite microstructure, reveals angular granite particles embedded within the matrix, with localized clusters and good bonding. Figure 6c, corresponding to 95% waste PTFE and 5% B_4_C, highlights the incorporation of boron carbide particles uniformly distributed throughout the matrix with evidence of strong interfacial bonding and minor voids. Finally, Fig. 6d, depicting the ternary composite (90% waste PTFE, 5% B_4_C, and 5% granite), demonstrates the most uniform structure, with dense reinforcement particles and minimal defects, suggesting enhanced mechanical and wear properties. As seen in Fig. 8(a-i), the image represents an elemental mapping of a composite based on PTFE and reinforced with hybrid granite and B_4_C, amounting to 5 vol% (PTFE 6 sample). Figure 8a shows the distribution of all the elements that make up the sample. Each section of the map illustrates the geographical distribution of specific components, such as carbon and fluorine which are found in PTFE (Fig. 8b and e, respectivly), boron which is found in B_4_C (Fig. 8c), and oxygen (Fig. 8d), magnesium (Fig. 8f), silicon (Fig. 8g), iron (Fig. 8h), and calcium (Fig. 8i) which are found in granite. On each map, brighter regions imply larger concentrations of the element in question, whilst darker parts suggest a lower component presence. This shows the quality of the reinforcement dispersion and any evidence of particle agglomeration. This mapping assists in evaluating the structural homogeneity of the composite.

### Density and porosity

The bulk density, relative density, and total porosity of PTFE-based composites were analyzed, with results summarized in Table 3. Also, the mean value, variance, and standard deviation of the measured bulk density are listed in Table 4. The theoretical densities of PTFE0, PTFE1, PTFE2, PTFE3, PTFE4, PTFE5, and PTFE6 samples are 2.200, 2.224, 2.216, 2.220, 2.232, 2.228, and 2.240 g/cm^3^, respectively. It is clear that the bulk density and total porosity of composites are slightly higher compared to PTEF, but the relative density is the opposite. The bulk density ranged from 2.227 to 2.251 g/cm³, relative density from 98.77 to 97.48%, and total porosity from 1.87 to 3.99%. This increase in the bulk density of PTFE by adding B_4_C and granite is due to their density being greater than that of PTFE. Moreover, a decrease in relative density and an increase in the total porosity might result from adding granite and B_4_C, which could disturb the PTFE matrix’s compactness and inhibit the particles from fitting as closely as pure PTFE. These results are in good agreement with those discussed in Ref^39^.

**Table 3:** Bulk density, relative density, and total porosity of sintered samples.

Sample name	Theoretical densities (g/cm³)	Bulk density (g/cm³)	Relative density (%)	Total porosity (%)
PTFE0	2.200	2.1730	98.081	1.227
PTFE1	2.224	2.1813	98.408	1.919
PTFE2	2.216	2.1807	98.249	1.592
PTFE3	2.220	2.1811	97.788	1.751
PTFE4	2.232	2.1826	97.950	2.212
PTFE5	2.228	2.1823	97.479	2.05
PTFE6	2.240	2.1835	98.081	2.521

**Table 4:** The mean value, variance, and standard deviation of the measured bulk density of PTFE0, PTFE1, PTFE2, PTFE3, PTFE4, PTFE5, and PTFE6 samples.

Sample name	Mean value	Variance	Standard deviation
PTFE0	2.1730	7.60 × 10^−6^	0.00028
PTFE1	2.1813	4.16 × 10^−8^	0.00020
PTFE2	2.1807	2.93 × 10^−7^	0.00054
PTFE3	2.1811	2.06 × 10^−7^	0.00045
PTFE4	2.1826	1.01 × 10^−7^	0.00032
PTFE5	2.1823	1.44 × 10^−7^	0.00038
PTFE6	2.1835	1.36 × 10^−7^	0.00037

### Thermal properties

**Fig. 9:**
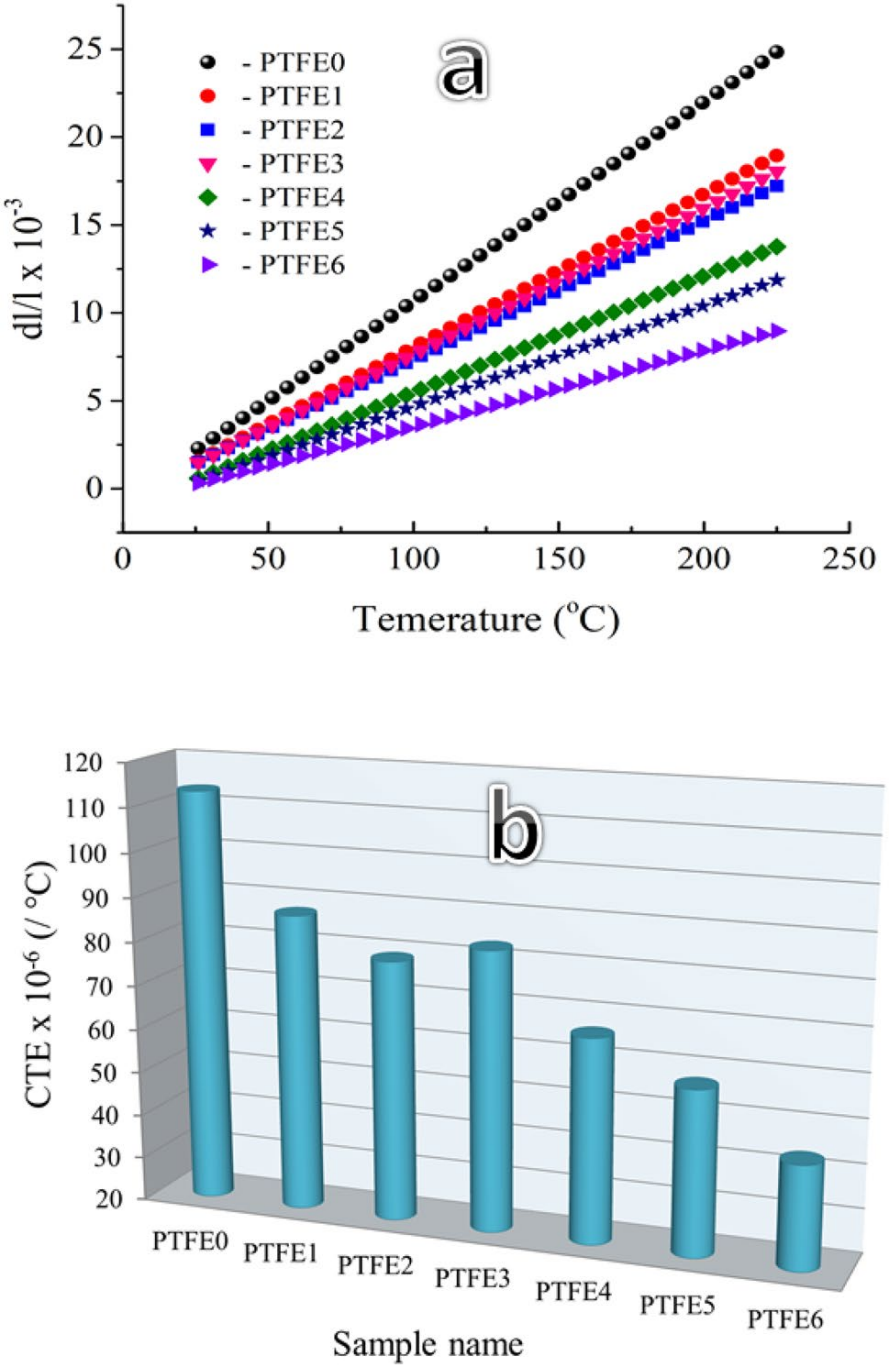
Thermal expansion and CTE value of PTFE and its composites.

It is undoubtedly important to evaluate the CTE for PTFE-based composites, as high thermal expansion can cause deformation of the composite material, leading to thermal failure in practical applications. Figure 9a displays the relative thermal expansion (dl/l) of PTFE0, PTFE1, PTFE2, PTFE3, PTFE4, PTFE5, and PTFE6 samples from 25 °C to 225 °C. In the same temperature range, the dl/l values for previous samples are 2.92 × 10⁻³ to 24.84 × 10⁻³, 1.57 × 10⁻³ to 18.95 × 10⁻³, 1.51 × 10⁻³ to 17.21 × 10⁻³, 1.49 × 10⁻³ to 18.05 × 10⁻³, 0.58 × 10⁻³ to 13.76 × 10⁻³, 0.44 × 10⁻³ to 11.85 × 10⁻³, and 0.33 × 10⁻³. Figure 9b demonstrates the variations in CTE of the samples estimated using the slope of the thermal expansion curve. The CTE value of PTFE is 113.4 × 10⁻⁶/°C, decreasing to 87.3 × 10⁻⁶, 78.9 × 10⁻⁶, 83.2 × 10⁻⁶, 66.2 × 10⁻⁶, 57.3 × 10⁻⁶, and 43.5 × 10⁻⁶/°C after adding 5 vol% granite, 5 vol% B_4_C, 2.5 vol% granite + 2.5 vol% B_4_C, 5 vol% granite + 2.5 vol% B_4_C, 2.5 vol% granite + 5 vol% B_4_C, and 5 vol% granite + 5 vol% B_4_C, respectively. The incorporation of B_4_C and/or granite into the PTFE matrix has been shown to reduce the CTE value, and this indicates that the interfacial bonding of PTFE-based composites is enhanced, the steric resistance of the PTFE matrix and reinforcements is increased. When subjected to external heat or force, the molecular chain movement is blocked. Stress transfer occurs, which decreases the expansion of the samples and improves the mechanical and tribological properties. The significant improvement in the CTE value of the PTFE matrix after adding reinforcements is due to the large difference in the CTE value of granite, B_4_C (≈ 7.8 and 5.3 × 10⁻⁶/⁰C), and the PTFE matrix (≤ 110 × 10⁻⁶/⁰C). These results agree well with Refs^40,41^.

### Mechanical properties

**Fig. 10:**
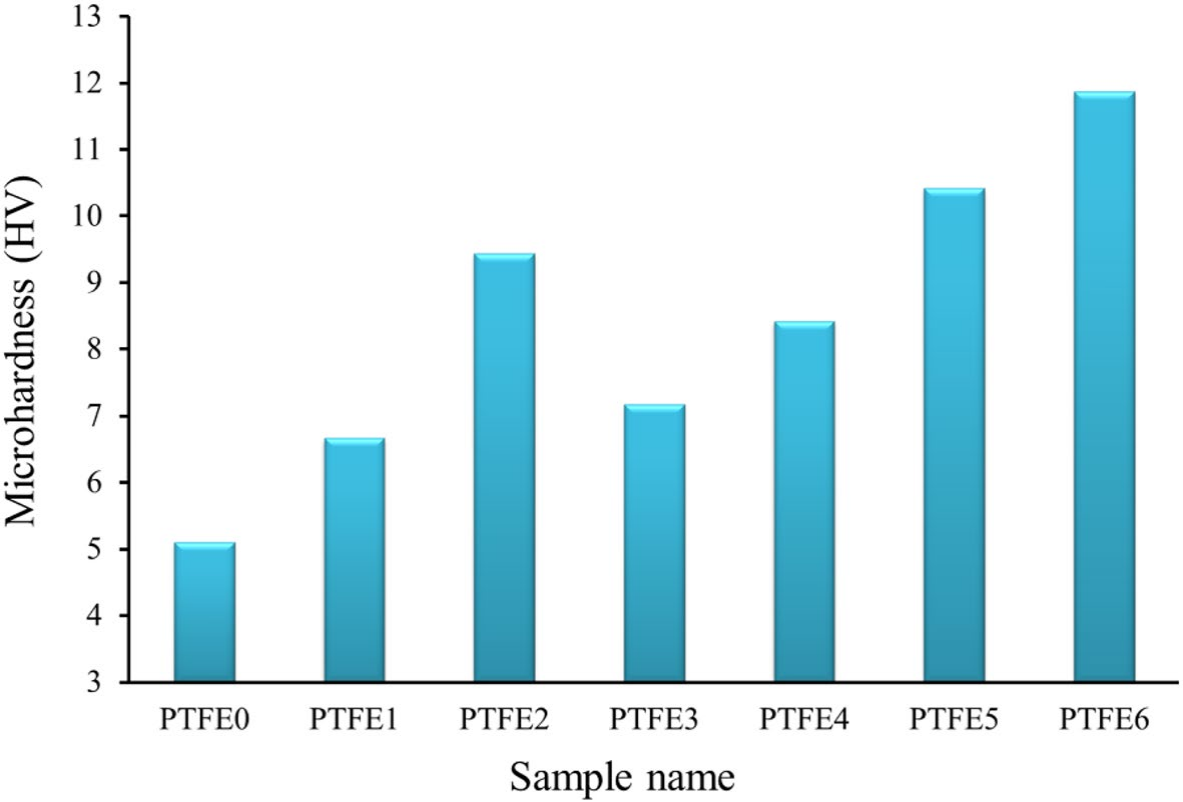
Effect of mono and hybrid reinforcement on microhardness of composite samples.

The mechanical properties of the PTFE composites, encompassing microhardness, compressive test, and elastic moduli, were thoroughly evaluated. The Vickers microhardness values of sintered PTFE0, PTFE1, PTFE2, PTFE3, PTFE4, PTFE5, and PTFE6 samples are shown in Fig. 10. Furthermore, the statistical analyses, including mean value, variance, and standard deviation, are tabulated in Table 5. Observing the results obtained in the figure, it is seen that the added mono and hybrid of granite and B_4_C volume percent are major reasons for the increases in microhardness values. The microhardness of PTFE1, PTFE2, PTFE3, PTFE4, PTFE5, and PTFE6 is 6.67, 9.44, 7.17, 8.42, 10.42, and 11.87 Hv, respectively, which improved by about 30.53%, 84.74%, 40.31%, 64.77%, 103.91%, and 132.29% compared to the PTFE sample (5.11 HV).

**Table 5:** The mean value, variance, and standard deviation of the measured microhardness of PTFE0, PTFE1, PTFE2, PTFE3, PTFE4, PTFE5, and PTFE6 samples.

Sample name	Mean value	Variance	Standard deviation
PTFE0	5.11	0.00032	0.018
PTFE1	6.67	0.00020	0.014
PTFE2	9.44	0.00012	0.011
PTFE3	7.17	0.00052	0.023
PTFE4	8.42	0.00028	0.017
PTFE5	10.42	0.00048	0.022
PTFE6	11.87	0.00012	0.011

**Fig. 11:**
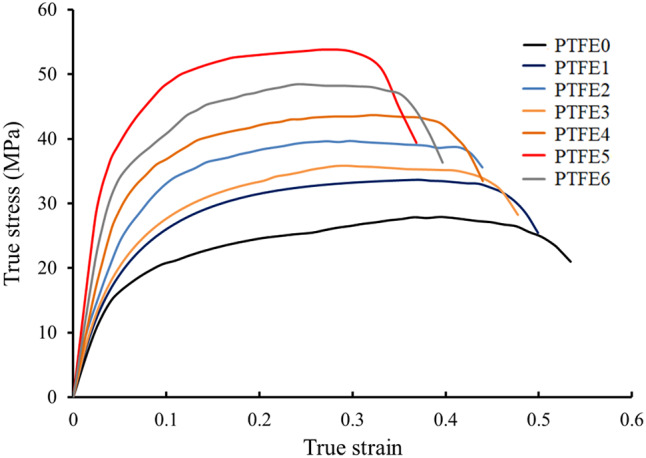
Compressive stress versus strain curve of PTFE and its composites.

**Fig. 12:**
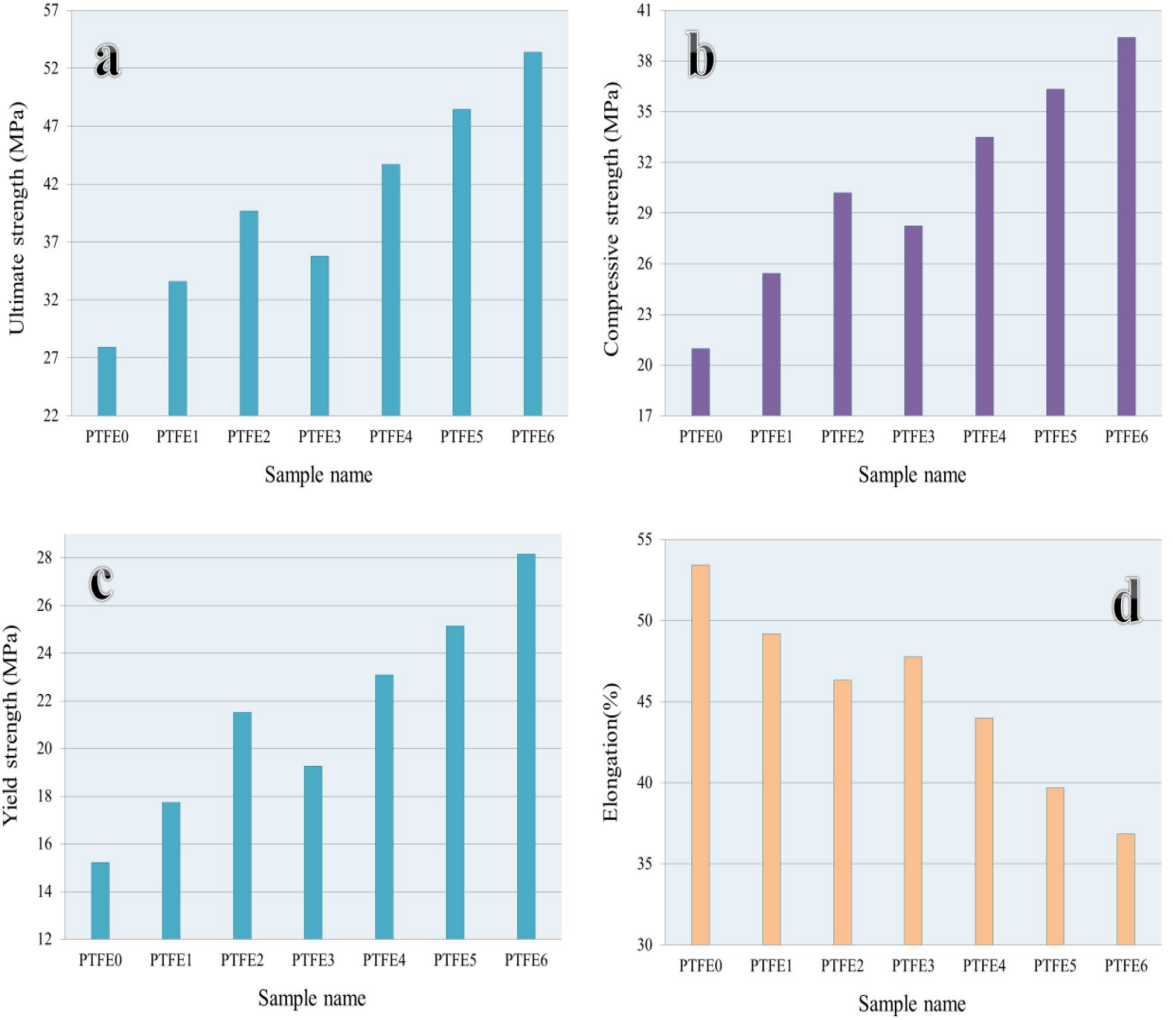
(**a**) Ultimate strength, (**b**) compressive strength, (**c**) yield strength, and (**d**) elongation of PTFE and its composites.

The compressive stress-strain curves of sintered PTFE and its composites are shown in Fig. 11. The ultimate strength, compressive strength, yield strength, and elongation are calculated from previous curves as shown in Fig. 12. As seen in earlier figures, adding the mono and hybrid ceramics volume percentages leads to remarkable increases in ultimate strength, compressive strength, and yield strength while elongation decreases.

For the PTFE1 sample, the ultimate strength, compressive strength, yield strength, and elongation are 27.91 MPa, 20.98 MPa, 15.22 MPa, and 35.42% and after adding reinforcements, all strengths increase up to 53.4, 39.40, and 28.14 MPa, respectively. In contrast, elongation decreases up to 36.85% for the PTFE6 sample.

**Fig. 13:**
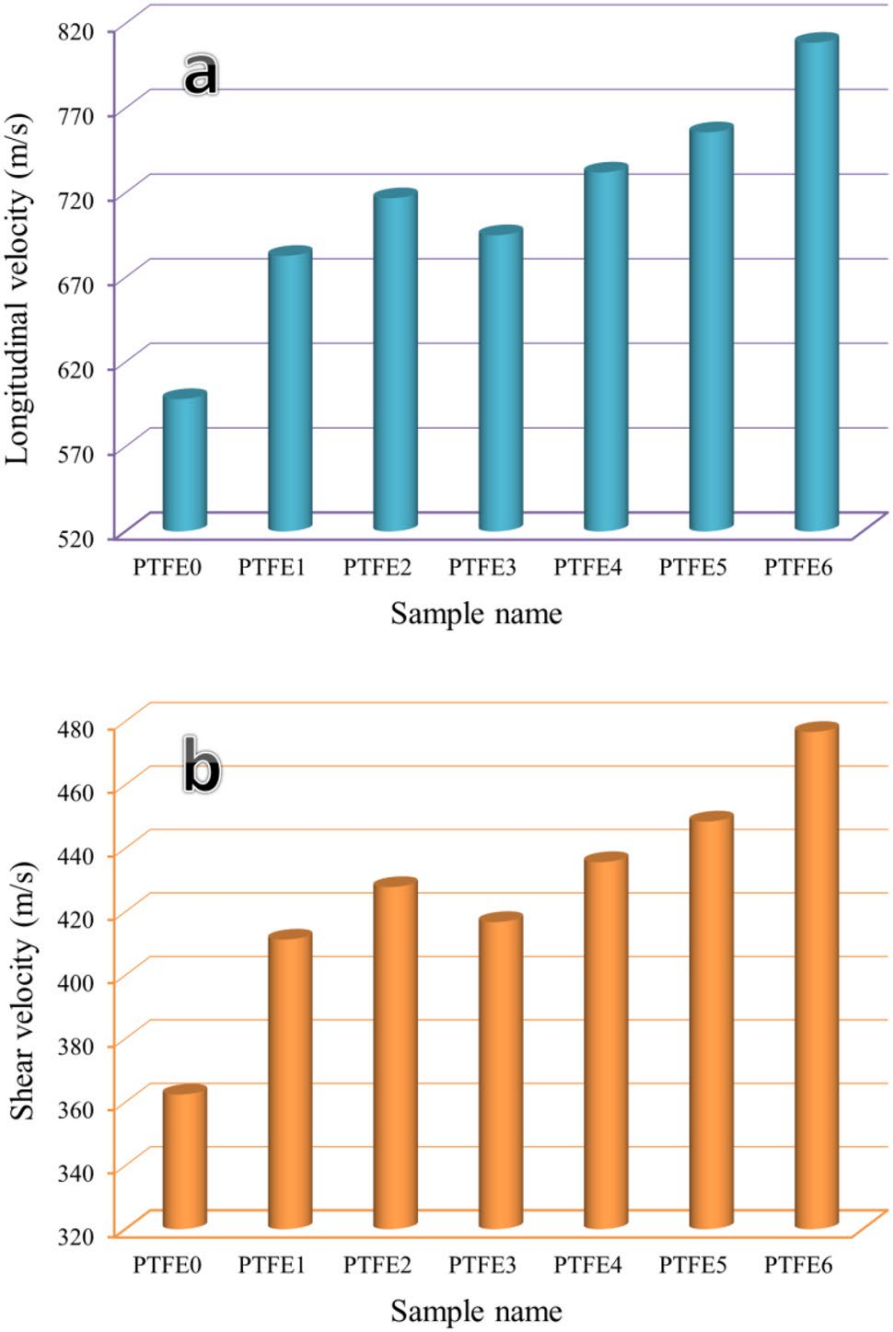
Effect of mono and hybrid reinforcement on ultrasonic velocities, i.e., (**a**) longitudinal velocity and (**b**) shear velocity of prepared samples.

Figure 13 displays the ultrasonic velocities results measured using the ultrasonic technique, and the elastic moduli are summarized in Table 6 for the same previous sintered samples using the ultrasonic nondestructive technique. Moreover, the mean value, variance, and standard deviation of the measured ultrasonic velocities are listed in Table 7. The figure clearly illustrates that ultrasonic velocities and all elastic moduli follow a similar tendency to microhardness and strength, demonstrating considerable increases in their values by incorporating mono and hybrid reinforcements. The longitudinal velocity of PTFE0 to PTFE6 is 598.17, 682.82, 716.84, 695.01, 732.11, 755.81, and 808.74 m/s, respectively, while the shear velocity of the same samples is 362.45, 411.28, 427.83, 416.81, 435.70, 448.50, and 476.71 m/s, respectively. The Young’s modulus of PTFE1, PTFE2, PTFE3, PTFE4, PTFE5, and PTFE6 is 894.16, 973.70, 921.19, 1012.75, 1075.12, and 1220.77 MPa, respectively, which improved about 29.44%, 40.96%, 33.35%, 46.61%, 55.64%, and 76.72% compared to the PTFE sample (690.78 MPa).

**Table 6:** The effect of added mono and hybrid reinforcement on the elastic moduli of the examined samples.

Sample name	Young’s modulus (MPa)	Longitudinal modulus (MPa)	Bulk modulus (MPa)	Shear modulus (MPa)	Possion ratio
PTFE0	690.78	777.52	492.04	285.47	0.2100
PTFE1	894.16	1013.99	646.12	367.87	0.2153
PTFE2	973.70	1117.23	719.27	397.96	0.2234
PTFE3	921.19	1050.41	672.62	377.79	0.2192
PTFE4	1012.75	1166.36	753.26	413.10	0.2258
PTFE5	1075.12	1242.92	805.26	437.67	0.2282
PTFE6	1220.77	1423.89	929.16	494.73	0.2338

**Table 7:** The mean value, variance, and standard deviation of the measured ultrasonic velocities of prepared samples.

Sample name	Mean value	Variance	Standard deviation
	Longitudinal velocity (m/s)		
PTFE0	598.17	9.075	3.013
PTFE1	682.82	5.971	2.444
PTFE2	716.84	3.991	1.998
PTFE3	695.01	4.795	2.190
PTFE4	732.11	11.740	3.426
PTFE5	755.81	1.868	1.367
PTFE6	808.74	1.929	1.389
Shear velocity (m/s)			
PTFE0	362.45	8.107	2.847
PTFE1	411.28	5.726	2.393
PTFE2	427.83	3.912	1.978
PTFE3	416.81	4.668	2.161
PTFE4	435.70	6.656	2.580
PTFE5	448.50	3.166	1.779
PTFE6	476.71	2.874	1.695

The enhancement of the mechanical properties of PTFE after the incorporation of mono and hybrid reinforcements of granite and B₄C may be ascribed to many synergistic causes. Firstly, granite and B₄C are inflexible, durable ceramic reinforcements that, when integrated into the pliable PTFE matrix, function as load-bearing agents adept at absorbing and transferring stress during mechanical loading. Reinforcements provide remarkable hardness and rigidity, substantially improving the composite’s compressive strength and elastic moduli. Hybrid reinforcement facilitates a more uniform stress distribution, thereby reducing stress concentrations within the PTFE matrix and enhancing overall mechanical performance^42,43^. Secondly, the homogenous distribution of granite and B_4_C reinforcements leads to a more sophisticated and uniform microstructure, significantly improving the material’s overall performance. This homogeneity inhibits particle aggregation, which may otherwise serve as vulnerabilities within the matrix, resulting in structural discrepancies. Consequently, the material demonstrates more uniform and dependable mechanical properties over its whole volume^44,45^. Finally, the use of hard reinforcements limits the mobility of PTFE chains, leading to a stiffer and mechanically stable configuration. This augmented rigidity elevates the material’s Young’s modulus and hardness, concurrently diminishing its propensity for creep and deformation under applied stresses, enhancing its long-term mechanical performance^46^. These findings are substantially consistent with those documented in Refs^47–49^.

### Tribological properties

**Fig. 14:**
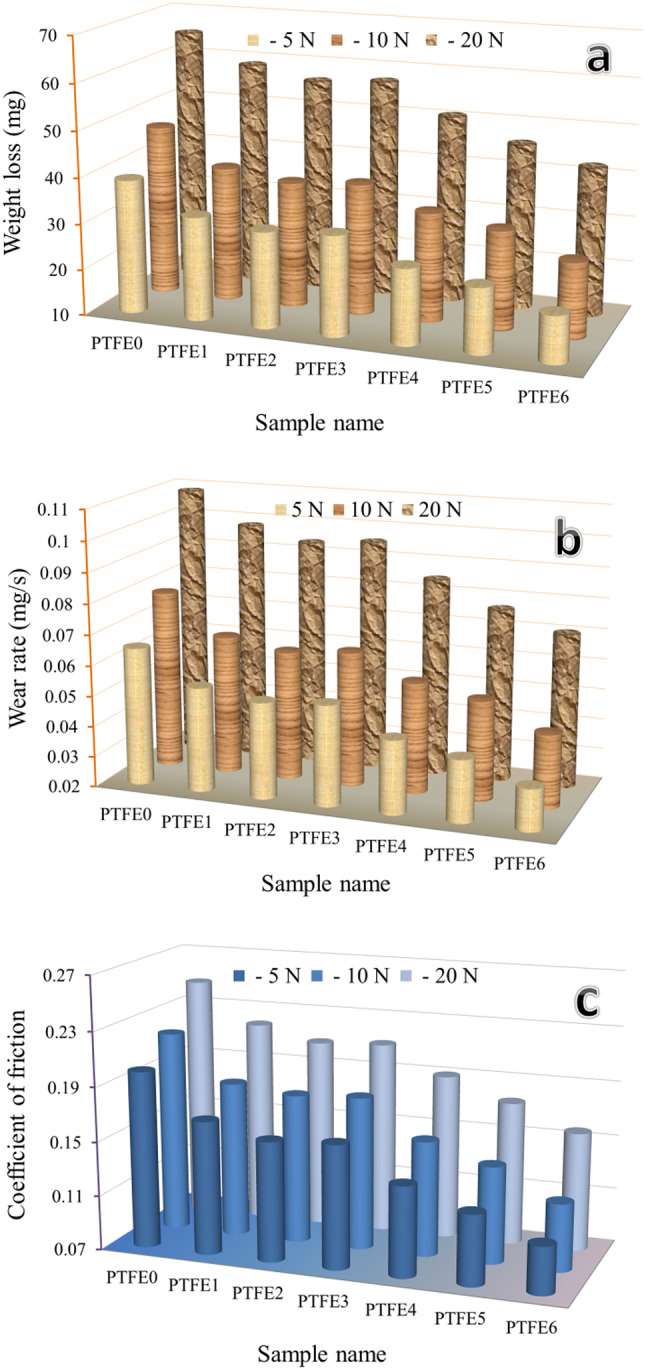
(**a**) Weight loss, (**b**) wear rate, and (**c**) coefficient of friction of all sintered samples.

The tribological performance of the PTFE-based nanocomposites was evaluated by measuring the weight loss, wear rate, and coefficient of friction (COF) under different loads (5, 10, and 20 N) and is visualized in Fig. 14. Furthermore, the statistical analyses of the measured wear rate at three different applied loads are listed in Table 8. Generally, the wear resistance and COF of the mono and hybrid composites decreased significantly with the addition of reinforcements and increased with increasing applied load; especially PTFE6 exhibited the lowest values across all loads, indicating smoother sliding behaviour. With a 5 N load, the unreinforced sample (PTFE0) had the highest wear rate (0.0650 mg/s). As the composite samples PTFE1-PTFE6 were added, the value dropped to 0.0541, 0.0514, 0.0528, 0.0440, 0.0402, and 0.0336 mg/s, which is a drop of 18.92%, 22.87%, 20.76%, 34.03%, 42.18%, and 49.64%, in that order. With the increased applied load to 20 for the same sample, the decreases in wear rate are 9.61%, 13.57%, 12.07%, 22.01%, 29.48%, and 35.18%. Likewise, for an applied load of 5 N, the COF values of the previous composite samples are 0.1681, 0.1581, 0.1607, 0.1363, 0.1217, and 0.1049, which decrease by about 15.99%, 21.01%, 19.69%, 31.88%, 39.20%, and 47.59% compared to the PTEF matrix (0.2001). The reduced COF values of composite samples are 11.83, 15.89, 15.01, 23.04, 29.34, and 36.52, respectively, when the applied load increases to 20 N. Generally, the wear performance of PTFE-based composites showed better abrasive wear performance. This can be attributed to the significant improvement in the mechanical properties of the prepared composites, which play a role in improving the wear resistance of the PTFE matrix^50,51^. Moreover, there is a reduction in the coefficient of friction and an increase in abrasion resistance when reinforced ceramics have a high hardness and thermal stability. The significant relationship between the increase in the microhardness of the composite based on the PTFE matrix and the decrease in wear rate and COF is illustrated by observing the Archard Eq. 11^52^.

11 $$\:Q=K\frac{W}{H}$$

*Q* is the wear rate, *K* is the wear coefficient, *W* is the applied load, and H is the microhardness of the sample.

Furthermore, increasing the applied load increases wear rate and COF, which induces plastic deformation on the subsurface as the counterface penetration depth increases. Unfortunately, moderate to severe wear ensues if the applied load exceeds the critical load. These results are consistent with many articles that studied the effect of high-hardness materials, such as ceramics, on the wear resistance of lower-hardness materials such as metals and polymers^53,54^.

**Table 8:** The mean value, variance, and standard deviation of the measured wear rate at different applied loads, i.e., 5, 10, and 20 N, of prepared samples.

Sample name	Mean value	Variance	Standard deviation
	Applied load of 5 *N*		
PTFE0	0.0650	9.01 × 10^−7^	0.00095
PTFE1	0.0541	8.02 × 10^−7^	0.00089
PTFE2	0.0514	4.01 × 10^−7^	0.00063
PTFE3	0.0528	5.80 × 10^−7^	0.00076
PTFE4	0.0440	4.41 × 10^−7^	0.00066
PTFE5	0.0402	5.02 × 10^−7^	0.00071
PTFE6	0.0336	9 × 10^−7^	0.00095
Applied load of 10 N			
PTFE0	0.0784	9.28 × 10^−7^	0.00096
PTFE1	0.0654	5.44 × 10^−7^	0.00074
PTFE2	0.0623	5.02 × 10^−7^	0.00071
PTFE3	0.0641	6.56 × 10^−7^	0.00081
PTFE4	0.0562	8 × 10^−7^	0.00089
PTFE5	0.0526	5.44 × 10^−7^	0.00074
PTFE6	0.0440	9.32 × 10^−7^	0.00097
Applied load of 20 N			
PTFE0	0.1085	8.22 × 10^−7^	0.00091
PTFE1	0.0981	4 × 10^−7^	0.00063
PTFE2	0.0938	3.81 × 10^−7^	0.00062
PTFE3	0.0954	6.36 × 10^−7^	0.00080
PTFE4	0.0846	6.56 × 10^−7^	0.00081
PTFE5	0.0765	5.44 × 10^−7^	0.00074
PTFE6	0.0703	6.76 × 10^−7^	0.00082

**Fig. 15:**
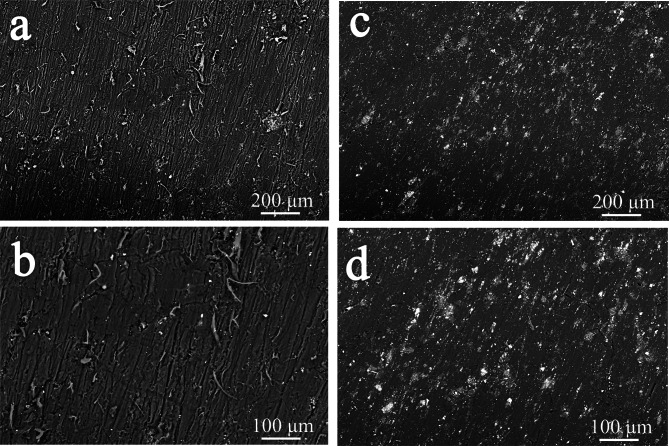
FESEM pictures at different magnification of wear tracks subjected to an applied load of 10 N for (**a** and **b**) PTFE0 and (**c** and **d**) PTFE6 samples.

FESEM was conducted on the worn surfaces of PTFE0 and PTFE6 samples to evaluate the wear processes under an applied load of 10 N, as seen in Fig. 15(a-d) Fig. 8a illustrates that virgin PTFE has many deep-striped grooves and wear detritus, presumably resulting from the furrow effect, indicative of significant abrasive wear. The buildup of wear debris exacerbates the degradation of the PTFE matrix (PTFE0), resulting in an elevated wear rate. The worn surfaces of PTFE composites (PTFE6) enhanced with granite and B₄C particles exhibit significant variations. The PTFE0 shows distinct abrasive grooves (Fig. 15a and b), but the PTFE6 presents a much smoother surface (Fig. 15c and d). These grooves are said to arise from friction against sharp, angular asperities on the hardened steel counter surface. The use of hybrid reinforcement seems to reduce surface damage, resulting in enhanced wear resistance.

## Conclusion

This article focuses on using PM technology to reuse PTFE waste as a matrix for composites reinforced with mono- and hybrid granite and B_4_C nanoparticles. The study concluded with the following findings:

- This method of recycling PTFE waste provides a more sustainable recycling opportunity and avoids the additional costs of disposing of this waste.
- By improving the sintering temperatures and using the appropriate pressure during the composites preparation, no changes in the composite content occurred, and XRD showed this.
- A slight decrease in the relative density after adding the reinforcements to reach 97.17 g/cm³ for the PTFE6 sample, a decrease of about 1.6% compared to the PTFE sample (98.77 g/cm³).
- The addition of reinforcements has a significant effect on thermal expansion, as the CTE value of the base sample is 113.41 × 10⁻⁶/°C and decreases to 43.50 × 10⁻⁶/°C (about 61.64%) after adding 5 vol% granite and 5 vol. B_4_C.
- Notable improvement in the mechanical properties of prepared composites and incorporated reinforcements. The maximum values of the microhardness and compressive strength of the PTFE6 sample were recorded, i.e., ~ 2.32 and 1.88 times, respectively, higher than those of the PTFE0 sample.
- The longitudinal and shear ultrasonic velocities of the sintered samples increased after adding granite and/or B_4_C, which led to an.
- Increase in the value of the elastic moduli. Compared to the base sample, the PTFE6 sample’s longitudinal and shear moduli increased to 80.22% and 70.95%, respectively.
- The wear resistance of the prepared samples improves by adding reinforcements, while increasing the applied load harms the wear resistance. For the applied loads of 5, 10, and 20 N, the wear rate for the PTFE sample decreased by about 48.35%, 43.80%, and 35.18% compared with PTFE0 of ceramic reinforcements.

## Data Availability

The datasets generated and/or analyzed during the current study are not publicly available because all data are presented in the article and therefore, there is no need to include raw data but they are available from the corresponding author upon reasonable request.

## References

[1] Xu, M. et al. Tribological properties of PTFE-based fabric composites at cryogenic temperature. *Friction* **12**, 245–257 (2024).

[2] Chen, L. et al. Mechanisms behind the environmental sensitivity of carbon fiber reinforced PTFE. *Friction* **12**, 997–1015 (2024).

[3] Wang, H., Liu, X., Liu, J., Wu, M. & Huang, Y. Tailoring interfacial adhesion between PBAT matrix and PTFE-modified microcrystalline cellulose additive for advanced composites. *Polymers* **14**, 1973 (2022). 35631855 10.3390/polym14101973 PMC9145506

[4] Liu, Z. et al. Synergistic modification of the tribological properties of PTFE with polyimide and Boron nitride. *Mater. Sci. Eng. A*. **864**, 144201 (2023).

[5] Zhang, Y., Kou, K., Zhang, S. & Ji, T. Improving thermal properties of ultrafine-glass-fiber reinforced PTFE hybrid composite via surface modification. *J. Polym. Res.* **26**, 188 (2019).

[6] Han, Y., Zheng, B., Chen, J. & He, Q. Study on the reinforcing effect of nano-SiO₂ filled PTFE composites. *AIP Adv.* **9**, 055217 (2019).

[7] Shi, J., Suo, S. & Zhang, Q. Effect of temperature on tribological properties of PTFE composites under oil lubrication. *IOP Conf. Ser. Earth Environ. Sci.* **719**, 022095 (2021).

[8] Ficici, F., Ozdemir, I., Grund, T. & Lampke, T. Tribological behavior of PTFE composites with bronze particles by Taguchi method. *J. Compos. Sci.* **8**, 398 (2024).

[9] Bandaru, A. K., Khan, A. N., Durmaz, T. & Alagirusamy, R. O’Higgins, R. M. Mechanical properties of multilayered PTFE composites through hybridization. *Constr. Build. Mater.* **374**, 130921 (2023).

[10] Deshwal, D., Belgamwar, S. U., Bekinal, S. I. & Doddamani, M. Role of reinforcement on PTFE composites: A review. *Polym. Compos.* **45**, 14475–14497 (2024).

[11] Li, Y., Chen, Y., Guo, Y., Bian, D. & Zhao, Y. Tribological behavior of PEEK/PTFE composites reinforced with carbon fibers. *Materials* **15**, 7078 (2022). 36295146 10.3390/ma15207078 PMC9605238

[12] Deshwal, D., Belgamwar, S. U., Bekinal, S. I. & Doddamani, M. PTFE composites: A comprehensive review. *Polym. Compos.* **45**, e28802 (2024).

[13] Rikhter, P. et al. Life cycle environmental impacts of plastics: A review. (2025) (In Press).

[14] Peretyagin, N. Y. et al. Microstructure and properties of B₄C composites reinforced by graphene. *Russ Eng. Res.* **40**, 94–96 (2020).

[15] Liu, Y. et al. Tribological properties of ptfe/kevlar self-lubricating composites. *Surf. Coat. Technol.* **363**, 236–244 (2019).

[16] Yan, Y., Du, J., Ren, S. & Shao, M. Prediction of PTFE composites using machine learning. *Polymers* **16**, 356 (2024). 38337245 10.3390/polym16030356 PMC10857071

[17] Fayyaz, O., Yusuf, M. M., Bagherifard, S., Montemor, M. F. & Shakoor, R. A. B₄C-reinforced Ni–P nanocomposite coating. *J. Mater. Res. Technol.* **20**, 2323–2334 (2022).

[18] Issa, S. A. M. et al. Hybrid nanocomposites from iron waste with nbc/granite. *Nanomaterials* **13**, 537 (2023). 36770498 10.3390/nano13030537 PMC9920841

[19] Omran, G. H., Radhi, N. S. & Abass, B. A. Al–PTFE graded materials by powder metallurgy. *FME Trans.* **52**, 57–67 (2024).

[20] Vasilev, A. P. et al. PTFE modified with carbon fibers, zro₂, sio₂, BN. *Polymers* **15**, 313 (2023).36679195

[21] Suh, J. & Bae, D. PTFE composites with graphene nanoplatelets by solid-state processing. *Compos. Part. B Eng.* **95**, 317–323 (2016).

[22] Sharma, S. K. et al. Powder metallurgy of Mg-based materials. *Nanomaterials* **15**, 92 (2025). 39852707 10.3390/nano15020092 PMC11767998

[23] Xu, Y. J. et al. Density effect of PTFE–Cu liners in shaped charges. *Strength. Mater.* **51**, 616–623 (2019).

[24] Wang, Y. & Monetta, T. SiC-reinforced mmcs: Preparation & properties. *J. Mater. Res. Technol.* **25**, 7470–7497 (2023).

[25] Youness, R. A. & Taha, M. A. Powder metallurgy for metal-based nanocomposites. *Egypt. J. Chem.* **64**, 7215–7222 (2021).

[26] Sajan, S. & Philip Selvaraj, D. A review on polymer matrix composite materials and their applications. *Mater. Today Pro*. **47**, 5493–5498 (2021).

[27] Taha, M. A., Youness, R. A. & Zawrah, M. F. Review on nanocomposites fabricated by mechanical alloying. *Int. J. Min. Metall. Mater.* **26** (9), 1047 (2019).

[28] Chao, M. et al. Thermal conductivity and properties of al₂o₃/ptfe. *World Sci.* **14**, 1950064 (2019).

[29] He, R., Chang, Q., Huang, X. & Bo, J. Graphene oxide on carbon fibers for PTFE composites. *J. Adhes. Sci. Technol.* **32**, 995–1004 (2018).

[30] Zhen, J. et al. High-temperature behavior of ptfe/mos₂ composites. *Lubricants* **11**, 312 (2023).

[31] Shi, Y. et al. CNF-filled PTFE composites: properties. *J. Appl. Polym. Sci.* **104**, 2430–2437 (2007).

[32] Youness, R. A. et al. Alumina/titania nanocomposites for bone repair. *Ceram. Int.* **50**, 48640–48654 (2024).

[33] Youness, R. A. et al. Hardystonite nanocomposites for bone regeneration. *Ceram. Int.* **51** (11), 14141–14159 (2025).

[34] Hessien, M. A. et al. Zn–Cr–Co spinel properties via microwave synthesis. *J. Inorg. Organomet. Polym.* 35, In press (2025).

[35] Abulyazied, D. E. et al. PVA/hydroxyapatite/magnesia/SiC hybrid composites. *Egypt. J. Chem.* **67**, 411–422 (2024).

[36] Youness, R. A. & Taha, M. A. Ceramic additives and osseointegration of Ni–Ti. *Ceram. Int.* **50**, 25434–25452 (2024).

[37] Si, J. et al. Microstructure and magnetic properties of novel powder cores composed of iron-based amorphous alloy and PTFE. *Mater. Sci.* **57** (3), 1–13 (2022).

[38] Sato, K. et al. Deformation capability of poly(tetrafluoroethylene) materials: Estimation with X-ray diffraction measurements. *Polym. Test.* **113**, 107690 (2022).

[39] Zhou, Z. et al. BC/PTFE composite fiberboards: dielectric properties. *J. Mater. Sci. Mater. Electron.* **32**, 19506–19516 (2021).

[40] Liu, F. et al. Thermal expansion and properties of PTFE composites. *Compos. Sci. Technol.* **241**, 110142 (2023).

[41] Zimmermann-Ptacek, J. et al. h-BN-filled PTFE composites. *J. Appl. Polym. Sci.* **135**, 46859 (2018).

[42] Vasilev, A. P. et al. Mechanical and tribological properties of polytetrafluoroethylene modified with combined fillers: carbon fibers, zirconium dioxide, silicon dioxide and Boron nitride. *J. Polym.* **15**, 313 (2023). 10.3390/polym15020313 PMC9862799 36679195

[43] He, R. Improved mechanical properties of carbon fiber reinforced PTFE composites by growing grapheme oxide on carbon fiber surface. *Compo Interf*. **5**, 1–10 (2018).

[44] Xiao, W. & Ji, X. Nano fillers in PTFE composites: simulation and experiments. *J. Appl. Polym. Sci.* **138**, 51340 (2021).

[45] Valadez-Gonzalez, A. et al. Surface treatment effect in natural fiber composites. *Compos. Part. B Eng.* **30**, 309–320 (1999).

[46] Jin, W. et al. Enhancing high-frequency dielectric and mechanical properties of SiO_2_/PTFE composites from the interface fluorination. *Ceram. Int.* **48**, 28512–28518 (2022).

[47] Zimmermann-Ptacek, J. et al. Thermal and mechanical properties of h-BN/PTFE. *J. Appl. Polym. Sci.* **135**, 46859 (2018).

[48] Praveenkumara, J. et al. Synthetic fillers in hybrid polymer composites: review. *J. Text. Inst.* **113**, 1231–1245 (2022).

[49] Struchkova, T. S. et al. PTFE composites with carbon fibers and zeolite. *Lubricants* **10**, 4 (2022).

[50] Li, J. & Ran, Y. PTFE composites filled with glass and carbon fiber. *Mater. Sci. Technol.* **41**, 115–118 (2010).

[51] Zaki, M. Z. et al. Tribo-mechanical and thermal behavior of Cu hybrid nanocomposites. *Sci. Rep.* **14**, 17479 (2024). 39080290 10.1038/s41598-024-67173-9 PMC11289293

[52] Youness, R. A. & Taha, M. A. Role of ti₃alc₂ MAX phase in regulating biodegradation and improving electrical properties of calcium silicate ceramic for bone repair applications. *Sci. Rep.* **14**, 25811 (2024). 39468168 10.1038/s41598-024-74859-7 PMC11519508

[53] Taha, M. A., Gad, S. A. & Youness, R. A. Development of fe/sibr/si₃n₄/silica fume nanocomposites from recycled metal waste for industrial applications. *Sci. Rep.* **15**, 1529 (2025). 39789021 10.1038/s41598-024-81657-8 PMC11717956

[54] Vasilev, A. P. et al. Mechanical and tribological properties of polytetrafluoroethylene modified with combined fillers: carbon fibers, zirconium dioxide, silicon dioxide and Boron nitride. *Polymers* **15**, 313 (2023). 36679195 10.3390/polym15020313 PMC9862799

